# [FeFe]-hydrogenase maturation: H-cluster assembly intermediates tracked by electron paramagnetic resonance, infrared, and X-ray absorption spectroscopy

**DOI:** 10.1007/s00775-020-01799-8

**Published:** 2020-07-13

**Authors:** Brigitta Németh, Moritz Senger, Holly J. Redman, Pierre Ceccaldi, Joan Broderick, Ann Magnuson, Sven T. Stripp, Michael Haumann, Gustav Berggren

**Affiliations:** 1 Department of Chemistry, Ångström Laboratory, Molecular Biomimetics, Uppsala University, 75120 Uppsala, Sweden; 2 Physics Department, Molecular Biophysics, Freie Universität Berlin, 14195 Berlin, Germany; 3 Present Address: Department of Chemistry and Biochemistry, Montana State University, Bozeman, MT 59717 USA; 4 Physics Department, Biophysics of Metalloenzymes, Freie Universität Berlin, 14195 Berlin, Germany; 5 Present Address: Department of Chemistry, Ångström Laboratory, Physical Chemistry, Uppsala University, 75120 Uppsala, Sweden

**Keywords:** Metalloenzymes, [FeFe]-hydrogenase, H-cluster assembly, Time-resolved spectroscopy, Maturation intermediates

## Abstract

**Abstract:**

[FeFe]-hydrogenase enzymes employ a unique organometallic cofactor for efficient and reversible hydrogen conversion. This so-called H-cluster consists of a [4Fe–4S] cubane cysteine linked to a diiron complex coordinated by carbon monoxide and cyanide ligands and an azadithiolate ligand (adt = NH(CH_2_S)_2_)·[FeFe]-hydrogenase apo-protein binding only the [4Fe–4S] sub-complex can be fully activated in vitro by the addition of a synthetic diiron site precursor complex ([2Fe]^adt^). Elucidation of the mechanism of cofactor assembly will aid in the design of improved hydrogen processing synthetic catalysts. We combined electron paramagnetic resonance, Fourier-transform infrared, and X-ray absorption spectroscopy to characterize intermediates of H-cluster assembly as initiated by mixing of the apo-protein (HydA1) from the green alga *Chlamydomonas reinhardtii* with [2Fe]^adt^. The three methods consistently show rapid formation of a complete H-cluster in the oxidized, CO-inhibited state (Hox-CO) already within seconds after the mixing. Moreover, FTIR spectroscopy support a model in which Hox-CO formation is preceded by a short-lived Hred′-CO-like intermediate. Accumulation of Hox-CO was followed by CO release resulting in the slower conversion to the catalytically active state (Hox) as well as formation of reduced states of the H-cluster.

**Graphic abstract:**

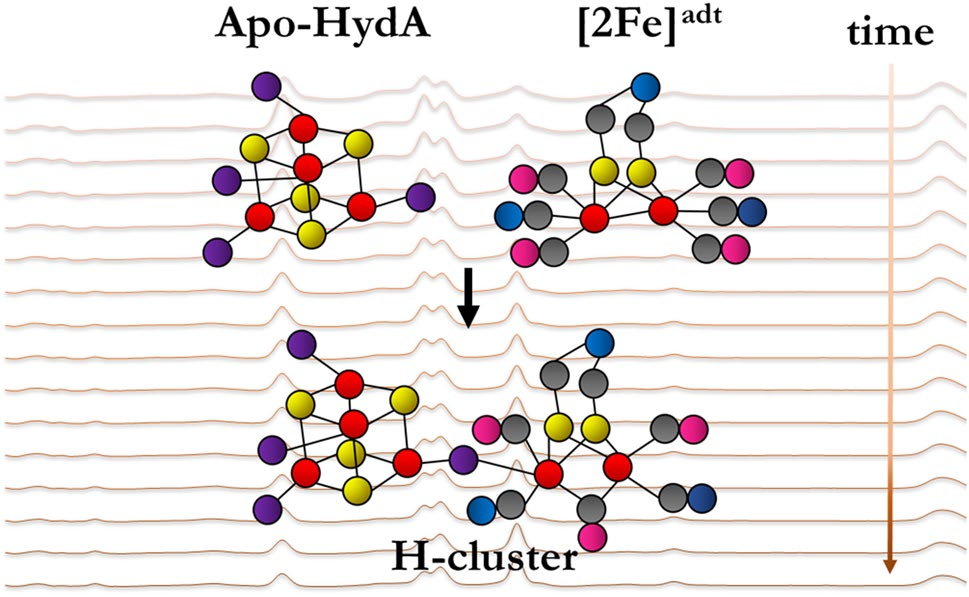

**Electronic supplementary material:**

The online version of this article (10.1007/s00775-020-01799-8) contains supplementary material, which is available to authorized users.

## Introduction

**Fig. 1:**
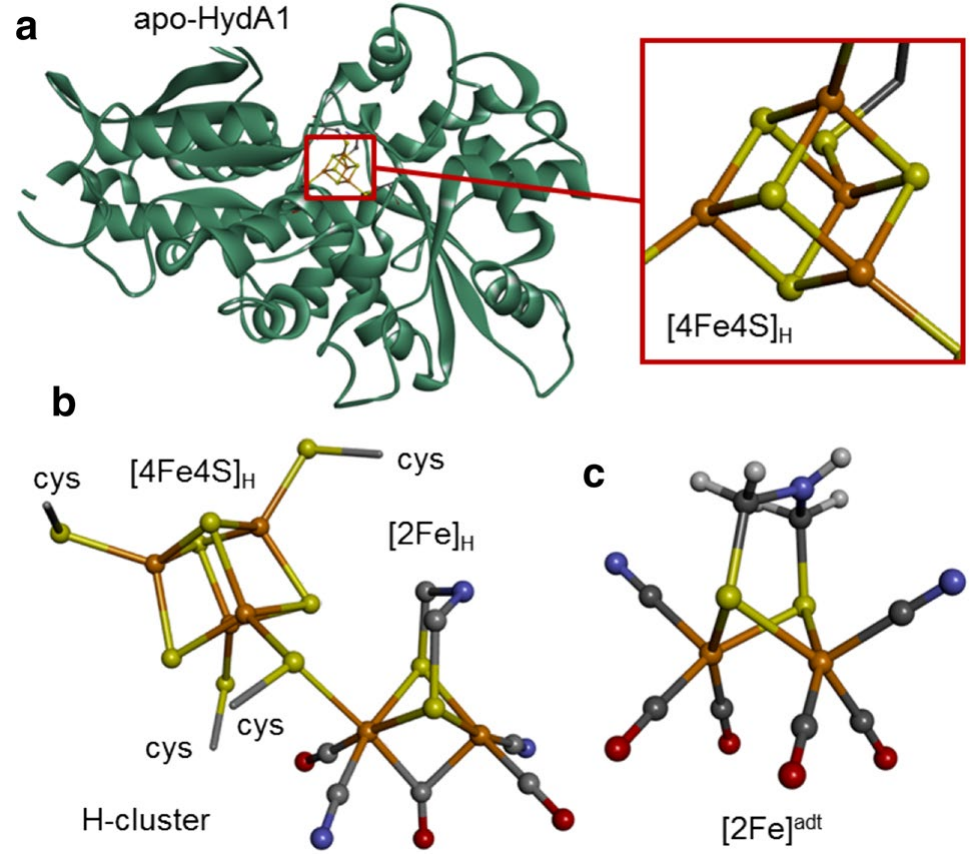
Crystal structures of iron components. Structures correspond to: **a** apo-HydA1 protein with the [4Fe–4S] cluster bound by four cysteines shown in magnification (PDB-ID 3LX4, ref [14]); **b** the complete H-cluster in the Hox state featuring a bridging CO ligand and an apical vacancy at the distal iron site of the diiron sub-complex (in a bacterial [FeFe]-hydrogenase, PDB-ID 4XDC, ref [15]); and **c** the synthetic diiron complex, [2Fe]^adt^, used for enzyme activation (color code: Fe, orange; O, red; N, blue; C, gray; H, white; protons were not resolved in protein structures; *cys* cysteine)

[FeFe]-hydrogenases utilize a unique cofactor denoted the H-cluster to catalyze the reversible interconversion of protons and electrons to molecular hydrogen with high turnover frequencies [1–3]. The H-cluster consists of a canonical iron–sulfur cluster ([4Fe–4S]_H_) fused with a dinuclear iron complex referred to as the diiron subsite ([2Fe]_H_). The latter organometallic complex is coordinated by carbonyl and cyanide ligands and bridged by an azadithiolate ligand (NH(CH_2_S)_2_, adt). The only protein ligand of the [2Fe]_H_ subsite is a bridging cysteine, connecting it to [4Fe–4S]_H_ (Fig. 1) [4–7]. Because [FeFe]-hydrogenases operate at ambient temperatures, near-neutral pH, and atmospheric pressure, these enzymes are excellent candidates for solar fuel production and therefore have inspired a wide range of biomimetic proton reduction catalysts [1, 8–10]. Different types of [FeFe]-hydrogenases are found in various organisms such as bacteria and green algae, but the active-site H-cluster is seemingly identical in all so-far characterized members of this enzyme family [2, 11]. In the following, we refer to the enzyme HydA1 from the green algae *Chlamydomonas reinhardtii*. In contrast to most bacterial [FeFe]-hydrogenases, HydA1 exclusively binds the H-cluster and no accessory iron–sulfur clusters [2, 12, 13], making it superior for spectroscopy on the active site, as employed in the present study.

The complex nature of the diiron subsite makes its synthesis in the cell dependent on a specialized enzymatic maturation machinery, which consists of at least three proteins, denoted HydG, HydE, and HydF [16]. In brief, HydG is a radical SAM enzyme, catalyzing the formation of CO and CN^−^ ligands that appear to form a mononuclear synthon during hydrogenase maturation [17–21]. HydE is also a radical SAM enzyme and most likely involved in the synthesis of the azadithiolate ligand, but the chemistry of HydE remains to be fully elucidated [22–24]. Finally, HydF seems to serve mainly as a scaffold protein, which in its holo-form carries a [2Fe]_H_ subsite-like pre-catalyst that is spontaneously transferred to apo-HydA1 containing [4Fe–4S]_H_ but lacking [2Fe]_H_ [14, 25, 26]. A combination of biomimetic synthetic chemistry, biochemistry, spectroscopy, and quantum chemical calculations has revealed that the pre-catalyst on HydF is strikingly similar, if not identical, to the synthetic complex [Fe_2_^I,I^(adt)(CO)_4_(CN)_2_]^2−^ ([2Fe]^adt^, Fig. 1c) [6, 27– 29]. Indeed, incorporating the [2Fe]^adt^ complex into either HydF or apo-HydA1 results in semi-synthetic forms of the two proteins, which are spectroscopically and biochemically indistinguishable from the native biological proteins [6, 15, 28, 29, 31].

Considering the final structure of the [2Fe]_H_ subsite and the structure of the [2Fe]^adt^ like pre-catalyst on HydF, the H-cluster assembly in vivo is a sophisticated multi-step process. Earlier H-cluster assembly studies have suggested that the reaction is initiated by the transfer of the pre-catalyst to the active site through a positively charged channel in the apo-protein [14, 32, 33]. In addition to the release of the pre-catalyst from HydF and its transfer to the binding site in HydA1, key steps must include the coordination of the bridging cysteine, release of a CO ligand, and diatomic ligand rearrangement, resulting in the formation of the so-called “inverted pyramid structure” of [2Fe]_H_ with a bridging CO ligand [34]. In vitro, quantitative incorporation of the diiron site into apo-HydA1 is readily achieved also without the maturation enzymes by mixing stoichiometric quantities of apo-HydA1 and the synthetic [2Fe]^adt^ complex. One equivalent carbon monoxide is released to form the functional H-cluster [15]. Protein film electrochemistry studies of H-cluster assembly have suggested that the H-cluster formation is at least a three-step process, with initial rapid formation of a [4Fe–4S]_H_/[2Fe]^adt^ adduct being followed by fusion of the two complexes and final CO release [33]. However, structural and spectroscopic information on the postulated intermediates is lacking. Thus, while the semi-synthetic approach has proven to be a powerful tool for elucidating the process of H-cluster formation [28, 32, 33], the details of the reaction between [4Fe–4S]_H_ and the [2Fe]^adt^ pre-catalyst still remain insufficiently understood.

To gain further insight into the assembly process, we probed H-cluster intermediates using a combination of freeze-quench electron paramagnetic resonance (EPR) and X-ray absorption (XAS) spectroscopy, as well as in situ Fourier-transform infrared (FTIR) spectroscopy. Our data show that the first intermediate that accumulates in significant quantities corresponds to the carbon monoxide-inhibited Hox-CO state of the H-cluster. FTIR data also show that Hox-CO, which accumulates on a tens of seconds timescale, may be preceded by a more reduced adduct of the cubane cluster and the diiron complex similar, or identical, to Hred′-CO. Fusion of the Fe(I)Fe(I) pre-catalyst and the [4Fe–4S]_H_^2+^ cluster on HydA1 accordingly results in a rapid electron transfer event to yield an Fe(I)Fe(II) state of the [2Fe]_H_ site retaining the fourth CO ligand. Slow release of the inhibiting CO ligand finally transforms the cofactor into the active Hox state. FTIR spectroscopy further identified one-electron reduced states (Hred, Hred′) accumulating in parallel to Hox.

## Materials and methods

### HydA1 apo-protein and diiron complex preparation

The chemicals were purchased from Sigma-Aldrich or VWR and used as received unless otherwise stated. The preparation of apo-HydA1 was carried out using previously published protocols with minor modifications [28, 35, 36]. (Et_4_N)_2_[Fe_2_(adt)(CO)_4_(CN)_2_] ([2Fe]^adt^]) was synthesized according to literature protocols with minor modifications and the integrity of the product was verified by FTIR spectroscopy [37–40]. Anaerobic work was performed in an MBRAUN glovebox ([O_2_] < 10 ppm). The purity of protein preparations was assayed by SDS-PAGE (10% minigels in a BioRad Mini-PROTEAN Tetra Cell system, stained with Page Blue, Thermo Fisher Scientific).

### EPR spectroscopy

100 µl aliquots of an apo-HydA1 protein solution (50 µM in 50 mM Tris–HCl, pH 8.0, 150 mM KCl) were mixed with 100 µl of a [2Fe]^adt^ solution (40 µM in 50 mM Tris–HCl, pH 8.0, 150 mM KCl), resulting in final concentrations of 25 µM protein and 20 µM [2Fe]^adt^ complex. The mixing was carried out inside of EPR tubes in the glovebox and the reaction mixture was either frozen immediately or incubated for a defined time period before freezing in an isopropanol cold well cooled by liquid nitrogen from the outside. After freezing, the samples were stored in liquid nitrogen until the EPR measurements. CW EPR measurements were carried out with an X-band EMX Micro EPR spectrometer (Bruker) using an ER049X SuperX microwave bridge in a Bruker SHQ0601 resonator equipped with a continuous-flow cryostat and an ITC 503 temperature controller (Oxford Instruments). The spectrometer was controlled by the Xenon software package (Bruker). Spectra were recorded with a 15 G modulation amplitude and a 100 kHz modulation frequency with 1 mW microwave power at 10 K at a microwave frequency of 9.38 GHz. Shown spectra represent the average of two magnetic field scans.

### FTIR spectroscopy

For attenuated total reflection Fourier-transform (ATR FTIR) spectroscopy, 1 μl apo-HydA1 protein solution (500 µM) was deposited on the three-reflection silicon crystal of an ATR cell (Smith Detections) in the beam path of a Tensor 27 FTIR spectrometer (Bruker) located in an anaerobic glovebox as described previously [41]. FTIR spectra (2 cm^−1^ spectral resolution, MCT detector at 80 kHz scanning velocity, 100 scans per spectrum) were recorded at ambient temperature (~ 24 °C), ambient pressure, and in the dark. The protein suspension was dried under 100% N_2_ gas and re-hydrated with buffer solution (100 mM Tris–HCl, MES, and PIPPS) in the humidified gas stream (aerosol), similar to our earlier reported procedures [41]. Cofactor insertion was started by application of 1 µl [2Fe]^adt^ solution (78 μM) on top of the protein film. Spectra in the CO/CN^−^ regime of the H-cluster were corrected for changes in protein concentration upon dilution with [2Fe]^adt^ solution (amide II band at 1545 cm^−1^) and fitted using the known IR signatures of Hox-CO, Hox, Hred, Hred′, and other H-cluster species [3].

### X-ray absorption spectroscopy

A solution (15 µl) of apo-HydA1 (~ 2 mM in 50 mM Tris–HCl, pH 8.0, 150 mM KCl) was mixed with a [2Fe]^adt^ solution (~ 2 mM in 50 mM Tris–HCl, pH 8.0, 150 mM KCl) in a 1:1 ratio, resulting in an iron concentration of ca. 6 mM in the samples. The mixing was followed by injection into Kapton-covered acrylic-glass XAS sample holders, after which the reaction mixture was either frozen immediately or incubated for increasing time periods before freezing inside the glovebox using an isopropanol cold well cooled by liquid nitrogen from the outside. After freezing, the samples were transferred and stored in liquid nitrogen until the XAS measurements. Two series of samples (22 samples in total) were prepared and analyzed. XAS at the Fe K-edge was performed at beamline KMC-3 at the BESSY-II synchrotron (Helmholtz Center Berlin, Germany; 250 mA top-up mode of the storage ring) as described earlier [42], using a setup including an Si[111] double-crystal monochromator, a 13-element energy-resolving Si-drift detector (RaySpec), and DXP-XMAP pulse-processing electronics (XIA). Samples were held at 20 K in a liquid-helium cryostat (Oxford). The energy axis of the monochromator was calibrated (accuracy ± 0.1 eV) using the K-edge spectrum of an iron metal foil (fitted reference energy of 7112 eV in the first derivative spectrum). The spot size on the samples was ca. 1 × 5 mm (vertical × horizontal) as set by a focusing mirror and slits. X-ray fluorescence spectra were collected using a continuous monochromator-scan mode (scan duration ~ 15 min). Up to seven scans to k = 18.2 Å^−1^ were averaged (1–2 scans per sample spot) for signal-to-noise ratio improvement. XAS data were processed (dead-time correction, background subtraction, normalization) to yield XANES and EXAFS spectra using our earlier described procedures and in-house software [42–45]. k^3^-weighted EXAFS spectra were simulated with in-house software and phase functions from FEFF9 (*S*_0_^2^ = 0.8) [46].

### Simulation of time courses

Parameter changes from XAS for increasing mixing periods (*t*) were simulated with a consecutive reaction scheme (Eq. 1) with three time constants (τ1, τ2, τ3), where the states were assigned according to the mixing protocols and data analyses to A, the initial apo-HydA1/[2Fe]^adt^ mixture; B, a rapidly formed (reduced) protein/complex adduct (possibly Hred′-CO); C, the Hox-CO state; and D, the Hox state of the H-cluster:

1 $$ A\xrightarrow{{\tau 1}}B\xrightarrow{{\tau 2}}C\xrightarrow{{\tau 3}}D. $$

The explicit formulas for the time dependences of species A, B, C, and D are given in the Supporting Information (Eq. S1).

## Results

The formation of the H-cluster starting from apo-HydA and [2Fe]^adt^ was monitored on a seconds to minutes timescale using a combination of EPR, FTIR, and X-ray absorption spectroscopy. The [FeFe]-hydrogenase from *Chlamydomonas reinhardtii* was prepared in a form containing only the [4Fe–4S] cluster (apo-HydA1) after heterologous expression in *E. coli* as previously described [35]. The H-cluster was then formed by mixing apo-HydA1 with the synthetic diiron complex [2Fe]^adt^ to yield fully active enzyme. The formation of assembly intermediates of the H-cluster was then monitored either in freeze-quench samples, which represented aliquots of apo-HydA1/[2Fe]^adt^ mixtures that were frozen at increasing time points after the initial mixing (EPR, XAS), or using sequential collection of spectra at room temperature (FTIR) after the initial protein/complex mixing, as described in the following.

### Redox reactions monitored by EPR

**Fig. 2:**
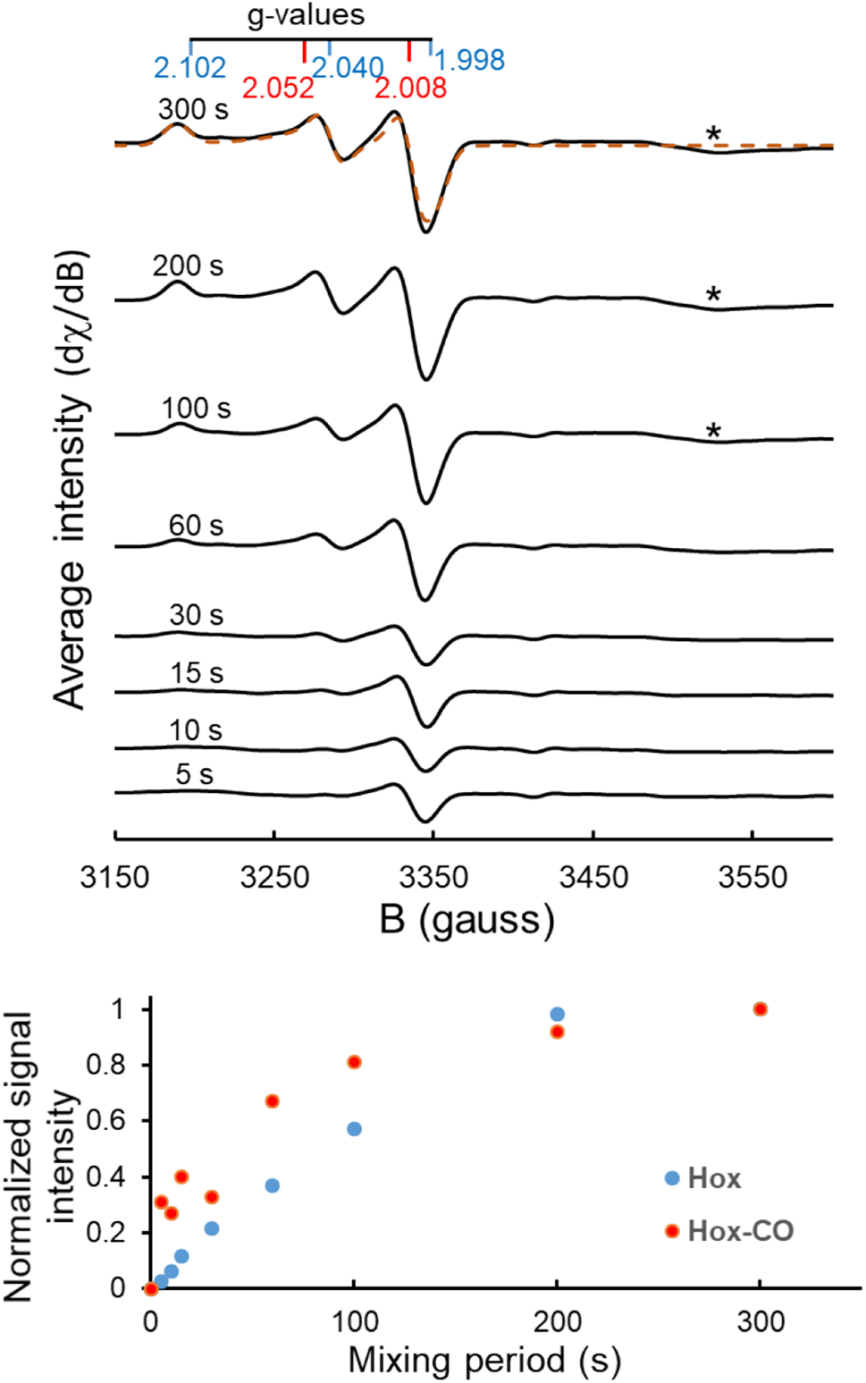
H-cluster assembly monitored by EPR spectroscopy. Top panel: spectra recorded for mixtures of apo-HydA1 and [2Fe]^adt^ incubated for increasing periods as indicated. Each spectrum represents the average of three replicates (spectra are vertically shifted for clarity; individual spectra are shown in Fig. S1). The *g* values for Hox (blue) and Hox-CO (red) are indicated (derived from spectral simulations, see Fig. S1; and a simulation of the final 300 s spectrum is overlaid, orange dashed line); a feature appearing at *g* = 1.91 (asterisks) is attributable to the formation of a reduced iron–sulfur cluster (see text) [47]. Bottom panel: relative signal intensities (symbols) of the Hox and Hox-CO states plotted vs. the incubation period (*t* = 300 s corresponding to 100% amplitude). The Hox and Hox-CO intensities were calculated through fitting a linear combination of their respective simulated spectra to the averaged experimental spectra shown in the top panel

EPR spectra were collected on three series of samples, each containing 20 µM [2Fe]^adt^ and 25 µM apo-HydA1. After the initial rapid mixing in the EPR tube, samples were incubated for increasing time periods (5–300 s, including the mixing process), then immediately frozen inside the glovebox using an isopropanol bath cooled by liquid nitrogen, the frozen tubes were transferred to the spectrometer cavity, and EPR spectra were recorded at 10 K (Fig. 2).

EPR spectra of the individual components, i.e., apo-HydA1 and [2Fe]^adt^ solutions, did not reveal any discernable signal attributable to the iron centers, demonstrating that the [4Fe4S] cluster in apo-HydA1 was in its EPR-silent oxidized state ([4Fe4S]_H_^2+^) and that the diiron compound comprised a likewise EPR silent Fe(I)Fe(I) center. In contrast, already at the first time-point after mixing of the two components (at about 5 s) an axial EPR signal was detectable. Its *g*-values (2.052 and 2.008), as derived from the spectral simulation (Fig. S1), indicated that the signal was attributable to the well-known Hox-CO state of the H-cluster, with an electronic configuration described as [4Fe–4S]_H_^2+^-[2Fe]_H_^(I,II)^ [2]. With increasing incubation periods, this signal increased in intensity until it reached a maximum after about 100–200 s (Fig. 2). After about 15 s, traces of a rhombic EPR signal (*g* = 2.102, 2.040, 1.998), attributable to the Hox state (Fig. S1), became discernable and continued to increase until the longest incubation period (Fig. 2) [2, 47]. At the end of the incubation period (300 s), the resulting EPR spectrum was best simulated with a mixture of approximately 65% Hox and 35% Hox-CO. On a minutes timescale a third species was visible in the EPR spectra as a small feature around *g* = 1.91, which also remained detectable until the longest incubation period (Fig. 2, asterisks). The latter signal is attributable to a reduced iron-sulfur cluster, i.e., to a [4Fe–4S]^+^ species in apo-HydA1 [26], albeit a double-reduced state of the complete H-cluster in holo-HydA1, such as Hsred (*g* = 2.076, 1.943, 1.868) or Hhyd (*g* = 2.07, 1.94, 1.88) with a [4Fe4S]_H_^+^ sub-complex, cannot be completely ruled out [47–49].

The EPR results indicate that the [2Fe]^adt^ pre-catalyst with an Fe(I)Fe(I) center becomes oxidized by one electron to form the Hox-CO state of the H-cluster (with an Fe(I)Fe(II) state of the [2Fe]_H_ subsite) [50], which is then converted to Hox with a similar redox state of [2Fe]_H_, by the release of one CO ligand from the diiron site. Already at the earliest accessible time point (~ 5 s), the only EPR-active species was Hox-CO, which suggests that cofactor oxidation occurs concomitantly with, or rather very rapidly following, the fusion of [4Fe–4S]_H_ and [2Fe]^adt^. In light of the oxidation states of the starting components, we presume that initially an EPR-silent, reduced H-cluster species is formed that may correspond to the Hred′-CO state [51]. This state is then oxidized to yield Hox-CO. The appearance of a signal attributable to a reduced [4Fe–4S]^+^ cluster in HydA1 suggests that the released electrons at least partially end up on the cubane cluster; alternatively, they can be disposed of via H_2_ formation. These considerations suggest that the H-cluster assembly is best described by a reaction sequence including at least three consecutive steps (Eq. 1 and XAS section below).

### Diatomic ligand changes followed by FTIR spectroscopy

**Fig. 3:**
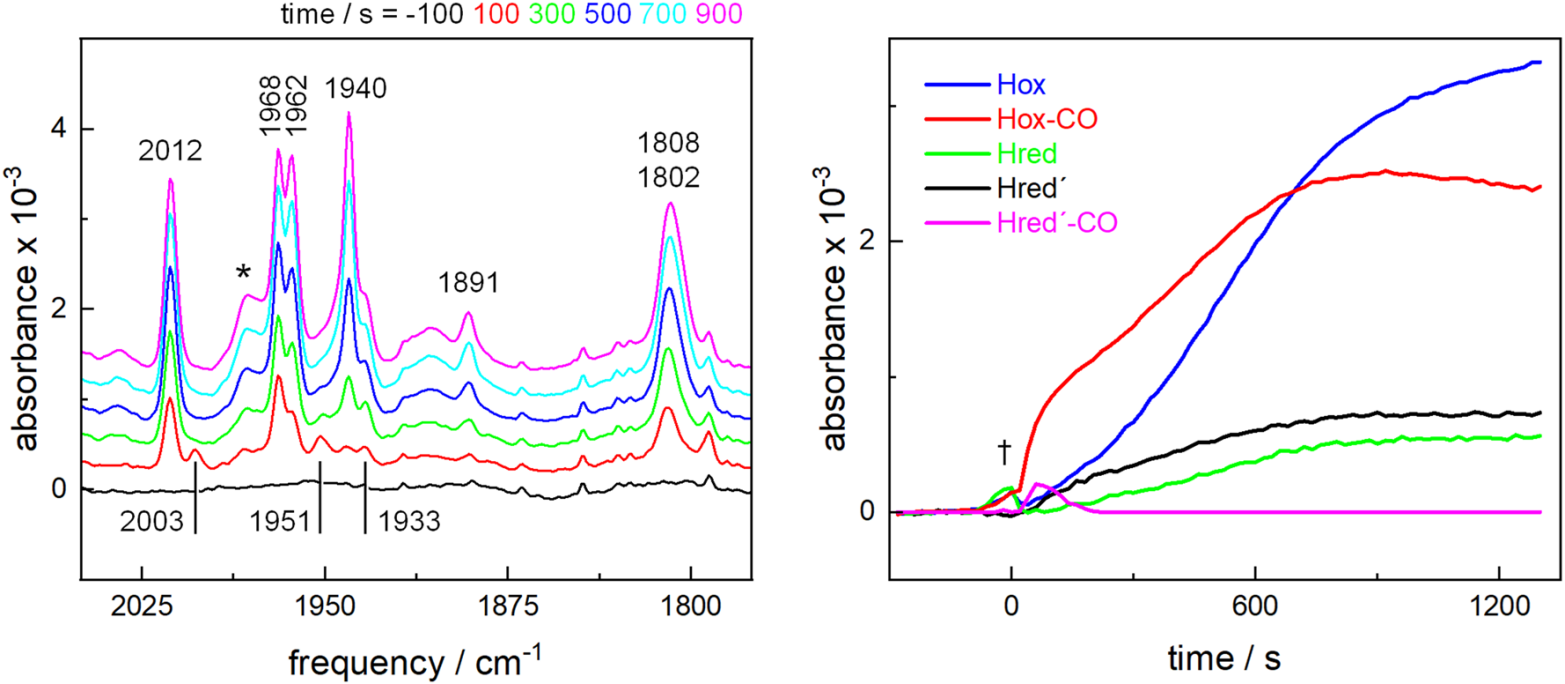
H-cluster assembly monitored by in situ ATR FTIR spectroscopy. [2Fe]^adt^ was added to a film of apo-HydA1 at around *t* = 0 s. Left panel: representative series of spectra for increasing incubation periods (in 100–200 s steps) showing H-cluster-specific bands in the CO vibrations region (marker bands: Hox-CO, 2012 cm^−1^; Hred′-CO, 2003 cm^−1^; Hox, 1940 cm^−1^; Hred′, 1933 cm^−1^; Hred, 1891 cm^−1^). *Background signal due to transient protein concentration changes upon injection of [2Fe]^adt^ (see Fig. S2). Right panel: changes of the intensities of marker IR bands of H-cluster states for increasing time periods after [2Fe]^adt^ addition. Band intensities were determined from the spectra in the left panel. †Transient contribution due to protein concentration changes upon injection of [2Fe]^adt^ (see Fig. S2), attributed to a vibrational band of water vapor

The H-cluster-specific stretching vibrations of the CO and CN^−^ ligands in the infrared region from about 2150 to 1750 cm^−1^ provide a specific probe for monitoring the reaction between [4Fe–4S]_H_ and [2Fe]^adt^. Earlier FTIR studies in transmission mode have shown that the broad IR bands of [2Fe]^adt^ in solution are replaced by much more narrow bands upon binding of the complex to [4Fe–4S]_H_ in [FeFe]-hydrogenase apo-protein [6, 15]. Thus, the detection of representative narrow CO/CN^−^ bands facilitates monitoring of the H-cluster assembly. Here, FTIR spectroscopy was performed on hydrated HydA1 protein films deposited on the surface of the crystal, in attenuated total reflection (ATR) mode and at room temperature [41]. At variance with the transmission experiment, the ATR approach enables monitoring of the time course of H-cluster assembly with a temporal resolution of about 1 s. HydA1 apo-protein (1 µL, 500 µM) was dried on the ATR crystal and re-hydrated in a stream of humidified N_2_ gas (aerosol). Then, a [2Fe]^adt^ solution (1 µL, 78 µM) was pipetted onto the film to start H-cluster generation. ATR FTIR spectra were recorded prior to and after the addition of the diiron complex (Figs. 3 and S2).

We focused the analysis of the FTIR spectra on the CO vibrations region because of the characteristic signatures and large signal intensities of these bands. The absence of any broad CO bands arising from the [2Fe]^adt^ complex in the spectra indicates that the starting species was not detectable, most likely due to its low concentration and immediate incorporation into the enzyme (Fig. 3). Instead, the characteristic infrared band signature of Hox-CO was the first clearly detectable species, which reached a maximum after about 800 s and decayed thereafter. Following a lag phase of approximately 200 s, the band signature of Hox started to appear in the spectra and continued to increase until the longest incubation period (1600 s). It should be noted that detection of CO bands on the ATR cell gives quantitative information on H-cluster formation no earlier than ~ 15 s after [2Fe]^adt^ addition because of the temporary dilution of the protein film, which regained its initial hydration level after about 60 s (Fig. S2). Two EPR-silent, one-electron reduced states, Hred and Hred′, appeared at prolonged incubation periods (Fig. 3), despite conditions expected to favor oxidized forms of the H-cluster (N_2_ gas stream, no reductant such as dithionite). No infrared bands of the two-electron reduced states Hsred or Hhyd were detectable in the spectra throughout the experiment, supporting the notion that the *g* = 1.91 signal in the EPR spectra is attributable to reduced apo-HydA1.

Accordingly, FTIR spectroscopy reveals an order of events during H-cluster assembly very similar to the corresponding EPR measurements. However, the time course of the appearance of the H-cluster species in the above IR data was considerably slower than that in the EPR experiments. This is potentially due to the lower [2Fe]^adt^ to apo-HydA1 ratio in the ATR FTIR experiment as compared to a near-stoichiometric ratio in the EPR samples, as well as diminished velocities of diiron binding and CO release and diffusion under the protein film versus solution conditions. To further probe the effect of relative [2Fe]^adt^ and apo-HydA1 concentrations, activation experiments were performed with a 100-fold lower [2Fe]^adt^ concentration (~ 0.8 µM). A lower amount of the diiron complex relative to the protein yielded significantly decreased H-cluster FTIR signals in the film, but increased the rate of Hox formation relative to Hox-CO (Fig. S3). Still, regardless of [2Fe]^adt^ concentration, the same order of the appearance of H-cluster species was observed. These findings, as well as the differing kinetics observed in the XAS data (below), suggested that the apparent velocities of the intermediate formation after the protein/complex mixing were determined by the sample conditions and did not represent the intrinsic reaction rates. Nevertheless, also the FTIR data support a model of consecutive Hox-CO and Hox state formation. Interestingly, a small amount of the Hred′-CO state of the H-cluster was detectable as a transient species at a low population for short (< 200 s) mixing periods (Fig. 3). This finding provides experimental support that rapid Hred′-CO formation precedes H-cluster oxidation resulting in Hox-CO.

Our present results show that under oxidizing conditions, that is, under an N_2_ atmosphere in the absence of an explicit reductant, H-cluster formation occurs spontaneously in solution and in protein films. Earlier protein-film electrochemistry studies of the H-cluster assembly reaction have shown that the oxidation state of [4Fe–4S]_H_ in apo-HydA1 is a critical factor for the fusion with the [2Fe]^adt^ pre-catalyst [33]. Thus, we repeated the ATR FTIR experiment in the presence of excess sodium dithionite as a chemical reductant (Fig. S4). The addition of [2Fe]^adt^ to apo-HydA1 under reducing conditions resulted in rather complex infrared spectra with contributions from the Hox, HoxH, and Hhyd states, but only insignificant proportions of Hred, Hox-CO, and Htrans Accordingly, H-cluster assembly does proceed also under reducing condition. However, the yield of the reaction, as estimated from the relative infrared band intensities, was about tenfold smaller than under non-reducing conditions (Fig. S5). Thus, the [4Fe–4S] cluster likely has to reside in its oxidized (2+) state for efficient [2Fe]^adt^ coupling.

### Structure and redox changes detected by XAS

XAS spectra at the Fe K-edge were collected on two series of samples containing a near-stoichiometric mixture of apo-HydA1 protein and the [2Fe]^adt^ complex (~ 1 mM each), which after mixing was loaded into the sample holders and incubated for increasing time periods (ca. 30–2000 s) prior to freezing in liquid nitrogen in the glovebox (Fig. S6). In addition, spectra were recorded of the individual starting components (apo-HydA1 and [2Fe]^adt^ solutions).

**Fig. 4:**
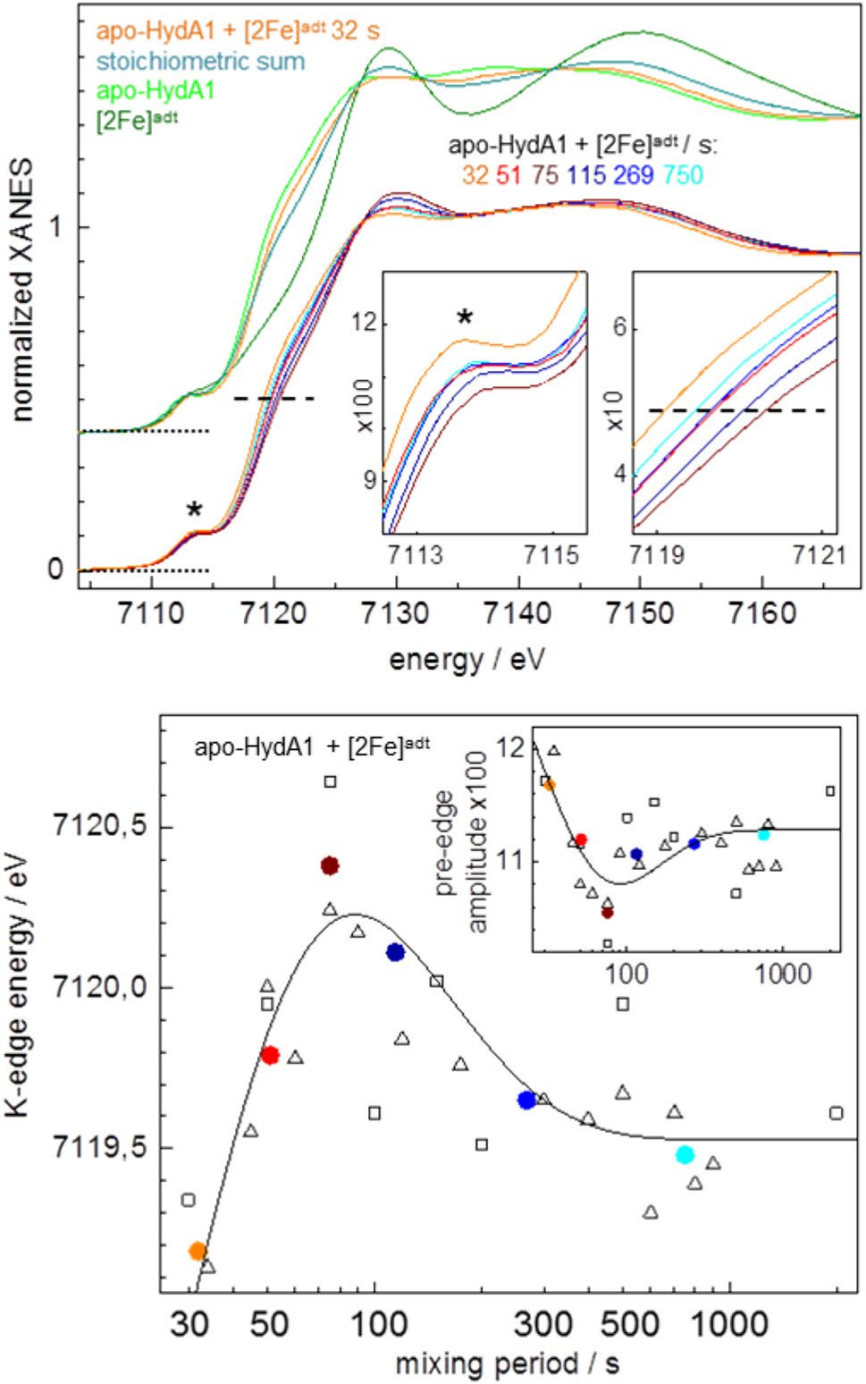
XANES spectra at the Fe K-edge. Top panel: spectra of apo-HydA1 protein and [2Fe]^adt^ complex solutions, the stoichiometric sum (4 × apo-HydA1 + 2x [2Fe]^adt^)/6, and the apo-HydA1 + [2Fe]^adt^ mixture after a mean incubation period of 32 s. Bottom: spectra of protein/complex mixtures after indicated mean incubation periods (derived from averaging of 2–4 spectra for comparable incubation periods, see Fig. S6). The insets show the pre-edge feature (left panel, asterisks) and the edge half-height (right panel, dashed lines) in magnification. Bottom panel: K-edge energies (at 50% XANES amplitude) and pre-edge amplitudes (at ~ 7113.5 eV) in the inset from spectra in Fig. S6 (black open squares and triangles denote data from two series of samples) and the top panel (colored solid circles denote data for respective mean spectra). The lines in the main and inset panels show simulations with a consecutive reaction scheme (Eqs. 1 and S1) with time constants (τ) of 5 s, 20 s, and 180 s

XANES spectra are shown in Fig. 4. The spectra of apo-HydA1 and [2Fe]^adt^ complex solutions were pronouncedly different and closely resembled earlier reported spectra, which indicated the structural integrity of the [4Fe–4S] cluster in the apo-HydA1 protein as well as of the [2Fe]^adt^ complex in solution (Fig. 4, top) [43, 51,52]. The spectra of the protein/complex mixtures even for the shortest incubation period (~ 30 s) differed from the stoichiometric sum of the apo-HydA1 and [2Fe]^adt^ solution spectra (Fig. 4, top). This finding suggested that already for ~ 30 s mixing time, the mean electronic and structural configurations of the iron species differ from the configurations in the individual complexes. For increasing mixing periods, the XANES spectra revealed first a ~ 1 eV increase in the K-edge energy, which was followed by a ~ 0.5 eV K-edge energy decrease for longer periods (Fig. 4, bottom). These effects were accompanied by amplitude changes of the pre-edge feature (~ 7113.5 eV). The initial K-edge energy increase was compatible with the oxidation of about one out of six iron ions in the H-cluster as well as with overall iron site symmetry changes. A simulation of the time course of the K-edge energy and pre-edge changes was feasible with a three-step reaction scheme (Eqs. 1 and S1) (Fig. 4, bottom, see caption). Notably, considerable scattering of the XANES parameters as observed over the time series is attributed to slight variations in the relative concentrations, mixing homogeneity, and incubation periods due to the difficult sample handling conditions, i.e., pipetting and mixing of microliter protein and [2Fe]^adt^ volumes in the XAS sample holders under anaerobic glovebox conditions.

**Fig. 5:**
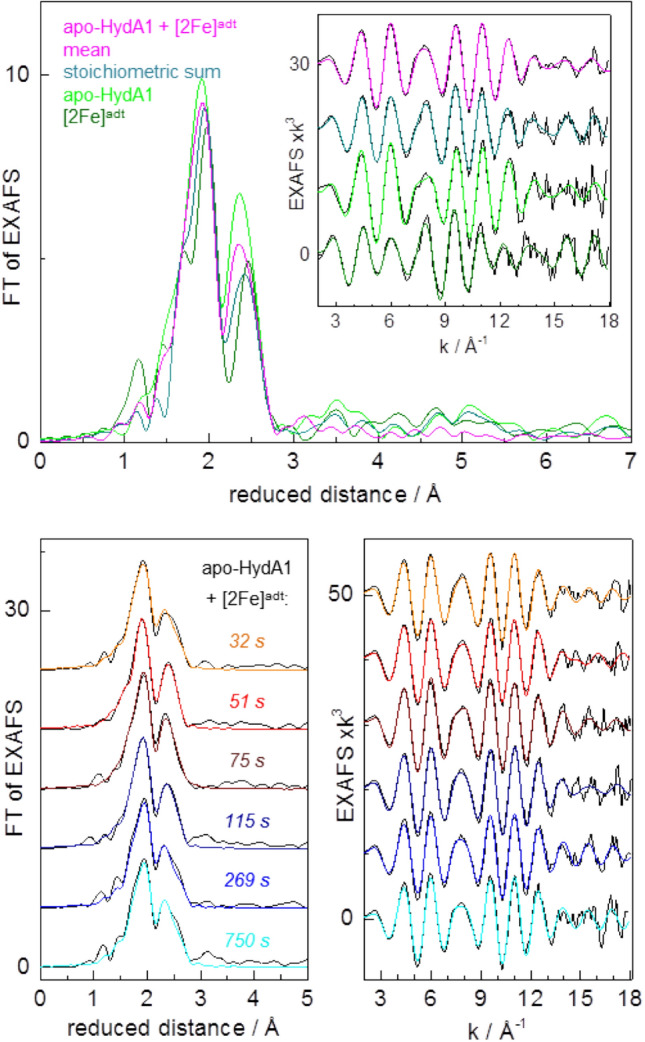
EXAFS analysis. Top panel: Fourier transforms (FTs) of EXAFS oscillations in the inset of apo-HydA1 protein and [2Fe]^adt^ solutions, the stoichiometric sum (see Fig. 4), and the apo-HydA1/[2Fe]^adt^ mixtures (mean spectrum over data for all incubation periods, Fig. S6). Inset: black lines, experimental data; colored lines, simulations with parameters in Table S1. Bottom panel: FTs in the left panel of EXAFS oscillations in the right panel (black lines, experimental data; colored lines, simulations with parameters in Table S1 and Fig. 6) for indicated mean mixing periods

**Fig. 6:**
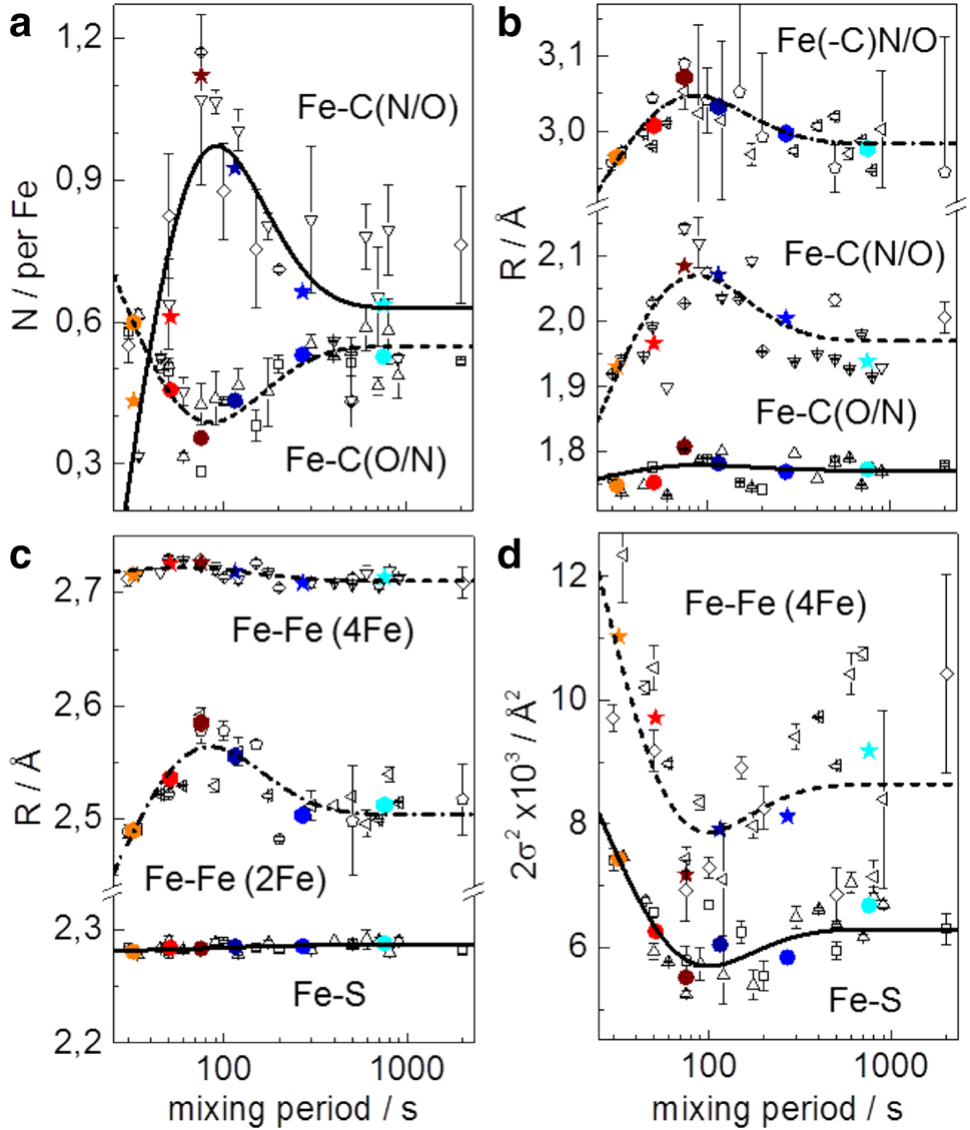
EXAFS simulation parameters. N, coordination number (**a**); R, interatomic distance (**b** and **c**); 2σ^2^, Debye–Waller factor (**d**) of indicated interactions; 4Fe and 2Fe denote parameters attributable to the [4Fe–4S] or diiron sites. Data correspond to spectra of apo-HydA1/[2Fe]^adt^ mixtures in Fig. S6 and Fig. 4 (open black symbols denote two series of samples; error bars represent variations for four different EXAFS fit approaches, Table S1; colored solid symbols denote averaged data for mean mixing periods, Fig. 5). The lines show simulations (Eqs. 1 and S1) with time constants as in Fig. 4 (5 s, 20 s, 180 s)

Changes in the structural features of the iron sites were addressed by EXAFS analysis. The EXAFS spectrum of apo-HydA1 (Fig. 5) as well as the respective Fe–S bond lengths, Fe–Fe distances, and coordination numbers from the spectral fit (Table S1) were close to the expected values and very similar to earlier determined data [52], which proved that the [4Fe–4S] cluster in apo-HydA1 was intact. The EXAFS of the [2Fe]^adt^ complex in solution showed the expected numbers of iron ligands as well as similar Fe–S and Fe–Fe distances as in the crystal structures, but slightly longer mean Fe–C(N/O) bond lengths (Table S1). The overall similar EXAFS spectra of the apo-HydA1/[2Fe]^adt^ mixtures (Fig. S6) suggested minor mean structure changes at the iron sites. Still, the mean EXAFS spectrum of the mixtures differed from the stoichiometric sum of the apo-HydA1 and [2Fe]^adt^ solution spectra (Fig. 5). It was well simulated with Fe–C(N/O) and Fe–S bond lengths and Fe–Fe distances (Table S1), which closely resembled the parameters from earlier EXAFS data and crystal structures of [FeFe]-hydrogenase holo-proteins (HydA1 and CPI enzymes) with a complete, oxidized (Hox) H-cluster [30, 44, 52, 54]. Simulations of the individual as well as the averaged EXAFS spectra for increasing apo-HydA1/[2Fe]^adt^ mixing periods revealed subtle, but consistent changes of the metrical parameters of the iron sites (Table S1, Figs. 5 and 6). A gain in the number of Fe–C(N/O) bonds per iron center and of longer (≤ 2.1 Å) at the expense of shorter (≤ 1.8 Å) Fe–C(N/O) bond lengths, a ~ 0.1 Å increase of the shorter Fe–Fe distance due to the diiron site, and a ~ 30% decrease of the Debye–Waller factors (2σ^2^) of the Fe–S bonds and Fe–Fe distances (~ 2.72 Å) in the [4Fe–4S] cluster for mixing periods up to ~ 75 s, as well as partial parameter change reversions for longer periods, were the major observed structure changes (Fig. 6). The main mean Fe–S and longer Fe–Fe distances, however, remained unchanged for increasing mixing periods. The kinetic behavior of the EXAFS parameter changes was reasonably described by the same time constants that accounted for the XANES changes (Fig. 6).

We interpret the changes of the XAS parameters in the protein/complex mixtures as follows. The XAS changes already at the shortest mixing period suggests the rapid formation of a (still reduced) apo-HydA1/[2Fe]^adt^ adduct, which leads to structural changes in particular at the [2Fe]^adt^ complex. The changes that follow include increased overall centro-symmetry and iron-ligand bond and Fe–Fe distance elongation at the diiron site as well as Fe–S and Fe–Fe distance homogenization in the [4Fe–4S] cluster. These findings are in good agreement with the transient formation of the Hox-CO state of the H-cluster, leading to two six-coordinated iron centers (and a µCO bridge) at the diiron site in Hox-CO as opposed to two five-coordinated iron sites in the [2Fe]^adt^ complex (lacking a µCO). Due to CO ligand release from the distal iron, Hox-CO decays slowly to Hox with five and six-coordinated iron centers (and a µCO) at the diiron site, which explains the partial reversion of the XAS parameter changes for long mixing periods. The ~ 0.1 Å Fe–Fe distance elongation as well as the further metrical parameters from EXAFS for ~ 75 s vs. ~ 750 s mixing are in good agreement with, e.g., the Fe–Fe distance change for the diiron site in Hox-CO vs. Hox, as found in geometry-optimized H-cluster structures from earlier quantum chemical calculations [55, 56].

## Discussion

**Scheme 1:**
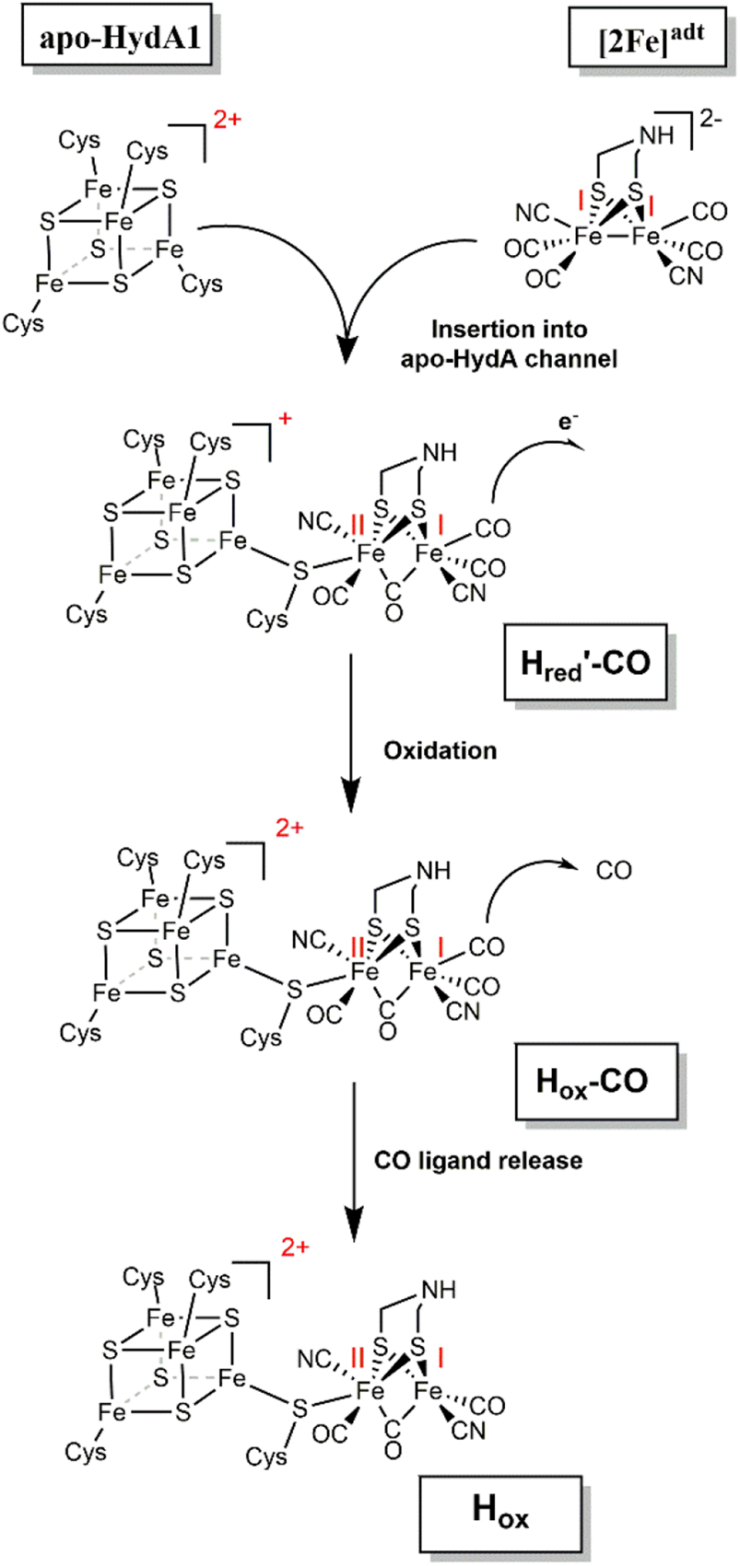
In vitro assembly of the H-cluster. Our present data (in combination also with earlier reports, refs [32, 33]) suggest at least three consecutive steps during the cofactor assembly. Fusion of the [4Fe4S]_H_ cluster and the [2Fe]^adt^ complex results in an early adduct, here assigned to the Hred′-CO state, followed by H-cluster oxidation to form Hox-CO, and final CO release yielding the active Hox state of the cofactor, which is the entry species into the catalytic H_2_-conversion cycle of [FeFe]-hydrogenases. Oxidation states of the [4Fe–4S]_H_ and [2Fe]_H_ components are indicated in red

The in vitro assembly of the H-cluster starting from purified [FeFe]-hydrogenase (apo-HydA1 carrying only the [4Fe–4S] cluster) and a synthetic diiron complex ([2Fe]^adt^) has been probed by EPR, FTIR, and X-ray absorption spectroscopy. The three methods consistently reveal that the first spectrally identified intermediate that accumulates to a significant extent already corresponds to the completely assembled H-cluster, which resides in the oxidized CO-inhibited state (Hox-CO). Accordingly, the fusion of the initial [4Fe–4S]^2+^ and Fe(I)Fe(I) complexes has to be accompanied by the loss of one electron to result in the [4Fe–4S]^2+^-[Fe(I)Fe(II)] configuration of the H-cluster in Hox-CO [14, 32, 33]. A more reduced H-cluster species, Hred′-CO, could be observed only as a minor species in the early FTIR spectra, concomitantly with Hox-CO, and no other reduced species were detected at short mixing periods. This is arguably because our three spectroscopic methods for technical reasons have allowed analysis of the apo-HydA1/2Fe^adt^ mixtures starting at 5–30 s after the mixing of the components. In addition, the absence of a corresponding EPR signal is expected due to the diamagnetic nature of such reduced intermediates. However, the apparent delay phase in the Hox-CO formation kinetics, as well as the lowered yield of H-cluster formation under chemically reducing conditions, supports the existence of at least one reduced earlier intermediate. We tentatively attribute this intermediate to a [4Fe–4S]_H_/[2Fe]^adt^ adduct with a similar total electron and CO ligand count as the two starting species. Such features and the FTIR data suggest that this transient species resembles the Hred′-CO state, corresponding to a [4Fe–4S]^+^-[Fe(I)Fe(II)] configuration of the H-cluster [51, 57]. Based on observations from biomimetic model chemistry, the oxidation of the diiron site is likely important for CO binding in the Fe–Fe bridging position, which is required to generate the catalytically relevant [2Fe]_H_ subsite configuration [34, 58, 59]. The reaction is potentially reversible at this stage, as reducing conditions in combination with a CO atmosphere were shown to result in [2Fe]^adt^ release [60, 61]. However, the reduced species does not accumulate, but instead is rapidly, relatively to the seconds timescale of these experiments, converted to Hox-CO via electron transfer to an external acceptor, thereby lowering the probability of the back-reaction. Under our conditions, cofactor oxidation presumably occurs via electron transfer between HydA1 proteins, as evidenced by the observation of reduced iron–sulfur cluster and H-cluster species in EPR and FTIR experiments, followed by proton reduction so that the excess electrons are released as H_2_ [34, 58, 59]. Finally, Hox-CO is converted by slow CO release to Hox with a similar redox state of the cofactor. Considering the low affinity of the reduced H-cluster states toward CO [33], it is noteworthy that the electron transfer event seemingly out-competes the CO release. The available experimental evidence therefore suggests a reaction sequence of in vitro H-cluster assembly comprising at least three consecutive steps (Scheme 1).

Our spectroscopic data for HydA1 protein were reasonably described by a three-step reaction sequence, but a more complex kinetic behavior was observed for protein film (ATR FTIR) versus solution (EPR and XAS) sample conditions. Different velocities of the three apparent cofactor assembly phases as observed in EPR, FTIR, and XAS indicate that the apparent reaction phases are not limited by the intrinsic reaction rates, such as entrance of [2Fe]^adt^ into the apo-HydA1 protein or chemical fusion of the two complexes, but rather are determined by the sample conditions. Along the same line, the higher protein concentration in the XAS compared to the EPR solution samples results in an accelerated H-cluster assembly, presumably mostly due to faster electron transfer for cofactor oxidation. In summary, the intrinsic chemical reaction between the four- and two-iron sub-complexes likely is much faster than the observed apparent velocities. We expect that optimization of the conditions for reaction steering, perhaps in combination with stopped-flow approaches, may facilitate detailed spectroscopic characterization also of the rapidly formed (reduced) H-cluster assembly intermediates in the future.

The spontaneous in vitro activation of [FeFe]-hydrogenase apo-enzymes just by the addition of the synthetic diiron complex in solution is still surprising when considering the underlying bond-breaking and bond-making chemistry. Obviously, the covalent connection between the four-iron and diiron sub-complexes is energetically downhill and therefore does not require any aid from a specific catalyst. In the cell, however, a complex maturation machinery, consisting at least of the three specific chaperones HydE, -F, -G, is required for H-cluster assembly. While the [4Fe–4S]_H_ cluster is readily assembled by the general iron–sulfur cluster housekeeping system, thus facilitating heterologous overexpression of [FeFe]-hydrogenase apo-proteins, most of the in vivo maturation effort is invested in the synthesis of the [2Fe] subsite complex. Recent studies have revealed that the CO, CN^−^, and adt ligands are synthesized and inserted with the help of HydE and HydG so that a diiron site precursor is formed on HydF [17, 18, 22, 23, 62], which is finally transferred to the apo-enzyme. The last step thus resembles the [2Fe]^adt^-promoted activation in vitro. Accordingly, the purpose of the inter-protein transfer of the diiron site from HydF to apo-HydA1 presumably is not to facilitate the fusion of the sub-complexes. Rather, HydF provides on-demand delivery of the precious complex as well as prevents its degradation under the in vivo conditions. Still, it should be noted that the capacity of HydF to serve as an electron acceptor is likely to facilitate the oxidation of the H-cluster (i.e., for Hox-CO formation) during the later stages of the reaction [28].

The efficient assembly of the native and functional H-cluster in apo-enzyme with the synthetic [2Fe]^adt^ complex, as well as the observed binding of many related diiron site mimics, offers the perspective for design and insertion of improved catalysts into protein scaffolds for hydrogen production applications both in vitro and in vivo [6, 8, 15, 35, 63, 64]. In parallel, our developing insight into the assembly mechanism of the biological catalyst may aid in the design of the next generation of biomimetic H_2_ producing synthetic materials.

## Electronic supplementary material

Below is the link to the electronic supplementary material.

Supplementary file1 (PDF 564 kb)

## Abbreviations

ATR FTIR: Attenuated total reflection Fourier-transform infrared
EPR: Electron paramagnetic resonance
EXAFS: Extended X-ray absorption fine structure
XANES: X-ray absorption near-edge structure
XAS: X-ray absorption spectroscopy
