# Supplementary material for: [FeFe]-hydrogenase maturation: H-cluster assembly intermediates tracked by electron paramagnetic resonance, infrared, and X-ray absorption spectroscopy
Source: J Biol Inorg Chem. 2020 Jul 13;25(5):777–88. doi: 10.1007/s00775-020-01799-8 (PMC7399679; doi:10.1007/s00775-020-01799-8)
Supplement: Supplementary file 1 — Supplementary file1 (PDF 564 kb) [file 775_2020_1799_MOESM1_ESM.pdf]

Supporting Information

[FeFe]-hydrogenase maturation: H-cluster assembly intermediates tracked by electron paramagnetic resonance, infrared, and X-ray absorption spectroscopy

Brigitta Németh[1, ‡], Moritz Senger[2, ‡ ‡], Holly J. Redman[1], Pierre Ceccaldi[1], Joan Broderick[3], Ann Magnuson[1], Sven T. Stripp[2], Michael Haumann[4], and Gustav Berggren[1, ✉]

[1]Department of Chemistry – Ångström Laboratory, Molecular Biomimetics, Uppsala University, 75120 Uppsala, Sweden

[2]Physics Department, Molecular Biophysics, Freie Universität Berlin, 14195 Berlin, Germany

[3]Department of Chemistry and Biochemistry, Montana State University, Bozeman, Montana 59717, USA.

[4]Physics Department, Biophysics of Metalloenzymes, Freie Universität Berlin, 14195 Berlin, Germany

[‡] Current address Department of Chemistry and Biochemistry, Montana State University, Bozeman, Montana 59717, USA.

[‡‡] Current address Department of Chemistry – Ångström Laboratory, Physical Chemistry, Uppsala University, 75120 Uppsala, Sweden

[✉]Correspondence to: gustav.berggren@kemi.uu.se

Table of Contents

Kinetic simulations 3

Table S1: EXAFS simulation parameters. 4

Fig. S1. Overview of simulated and experimental EPR spectra 5

Fig. S2. Protein film hydration monitored by ATR FTIR spectroscopy. 6

Fig. S3. Influence of the $[2Fe]^{adt}$ concentration on H-cluster assembly. 6

Fig. S4. H-cluster assembly under reducing conditions. 7

Fig. S5. Effect of reductant on H-cluster assembly (comparison). 7

Fig. S6. Fe XAS spectra of apo-HydA1/$[2Fe]^{adt}$ mixtures. 8

References 8

Kinetic simulations

Time courses of parameters from XAS were simulated on the basis of a consecutive 3-step reaction scheme (Eq. 1) and using Eq. S1 (Y1-4, scaling factors) for description of the formation of states C (i.e., **Hox-CO**) and D (i.e., **Hox**), with the respective time constants (τ) given in the text corresponding to the inverted rate constants ($\tau_i = k_i^{-1}$):

(S1)

$$A(t) = Y1\exp\left(\frac{-k1}{t}\right)$$

$$B(t) = Y2\left[\frac{k1}{(k2-k1)}\exp\left(\frac{-k1}{t}\right)+\frac{k1}{(k1-k2)}\exp\left(\frac{-k2}{t}\right)\right]$$

$$C(t) = Y3\left[\frac{k1k2}{(k2-k1)(k3-k1)}\exp\left(\frac{-k1}{t}\right)+\frac{k1k2}{(k1-k2)(k3-k2)}\exp\left(\frac{-k2}{t}\right)+\frac{k1k2}{(k1-k3)(k2-k3)}\exp\left(\frac{-k3}{t}\right)\right]$$

$$D(t) = Y4\left\{\frac{k1k2}{(k2-k1)(k3-k1)}\left[1-\exp\left(\frac{-k1}{t}\right)\right]+\frac{k1k2}{(k1-k2)(k3-k2)}\left[1-\exp\left(\frac{-k2}{t}\right)\right]+\frac{k1k2}{(k1-k3)(k2-k3)}\left[1-\exp\left(\frac{-k3}{t}\right)\right]\right\}$$

In the XAS data simulations it was assumed that species C and D were visible simultaneously in the data so that the sum of the C(t) and D(t) terms was included with individual scaling factors Y3 and Y4.

Table S1: EXAFS simulation parameters.[a]

sample	Fe-C(-N/O)	Fe-S	Fe-Fe	R_F [%]
	N [per Fe] / R [Å] / $2\sigma^2$ x10^3 [Å^2]			
[2Fe]^{adt}	2.0* / 1.81 (1.76) / 3$^{\&}$<br>2.0* / 2.75 / 3$^{\&}$<br>2.0* / 1.42 / 8$^{\S\#}$<br>1.0* / 2.02 (1.94) / 3$^{\&}$<br>1.0* / 2.98 / 3$^{\&}$<br>1.0* / 1.57 / 8$^{\S\#}$	2.0* / 2.29 (2.28) / 3	1.0* / 2.51 (2.51) / 3	14.2
apo-HydA1	-	4.1 / 2.29 (2.28) / 7	2.8 / 2.72 (2.72) / 10	13.5
apo-HydA1<br>+ [2Fe]^{adt}<br>mean	1.0* / 1.87 (1.86) / 5*<br>1.0* / 2.97 / 5*<br>1.0* / 1.34 / 5*$^{\#}$	3.3* / 2.29 (2.30) / 6	0.3* / 2.52 (2.55) / 2*<br>2.0* / 2.71 (2.69) / 9	10.5
32 s[b]	1.0* / 1.86 / 5*<br>1.0* / 2.98 / 5*<br>1.0* / 1.07 / 5*$^{\#}$	3.3* / 2.28 / 7	0.3* / 2.47 / 2*<br>2.0* / 2.72 / 12	14.1
51 s[b]	1.0* / 1.90 / 5*<br>1.0* / 2.99 / 5*<br>1.0* / 1.10 / 5*$^{\#}$	3.3* / 2.28 / 6	0.3* / 2.51 / 2*<br>2.0* / 2.72 / 10	11.4
75 s[b]	1.0* / 2.09 / 5*<br>1.0* / 3.02 / 5*<br>1.0* / 1.11 / 5*$^{\#}$	3.3* / 2.29 / 5	0.3* / 2.61 / 2*<br>2.0* / 2.73 / 8	10.2
115 s[b]	1.0* / 1.92 / 5*<br>1.0* / 3.00 / 5*<br>1.0* / 1.09 / 5*$^{\#}$	3.3* / 2.28 / 6	0.3* / 2.55 / 2*<br>2.0* / 2.72 / 9	13.3
269 s[b]	1.0* / 1.88 / 5*<br>1.0* / 2.98 / 5*<br>1.0* / 1.08 / 5*$^{\#}$	3.3* / 2.28 / 7	0.3* / 2.48 / 2*<br>2.0* / 2.71 / 10	14.2
750 s[b]	1.0* / 1.87 / 5*<br>1.0* / 2.98 / 5*<br>1.0* / 1.07 / 5*$^{\#}$	3.3* / 2.28 / 7	0.3* / 2.49 / 2*<br>2.0* / 2.71 / 10	15.0

[a]Data correspond to EXAFS spectra in Fig. 5. N, coordination number; R, interatomic distance; $2\sigma^2$, Debye-Waller factor; R_F, error sum calculated for reduced distances of 1-3 Å. [b]Data for averaged spectra of apo-HydA1/[2Fe]^{adt} mixtures (Figs. 5A and 5B). Fit restraints: *fixed parameter, $^{\&,\S}$parameter coupled to yield the same value for different shells, $^{\#}$parameters of a multiple-scattering shell of C(N/O) ligands. Distances from crystal structures of the [2Fe]^{adt} complex and of [FeFe]-hydrogenase apo or holo (oxidized) protein are given in parentheses [1, 2]. Data in Fig. 6 stem from a similar fit approach as for the mean apo-HydA1 + [2Fe]^{adt} spectrum, using a variable or fixed E_0 value in combination with a shorter or longer mean Fe-C(-O/N) bond length in the simulations of the individual EXAFS spectra of the protein/complex mixtures (Fig. S6).

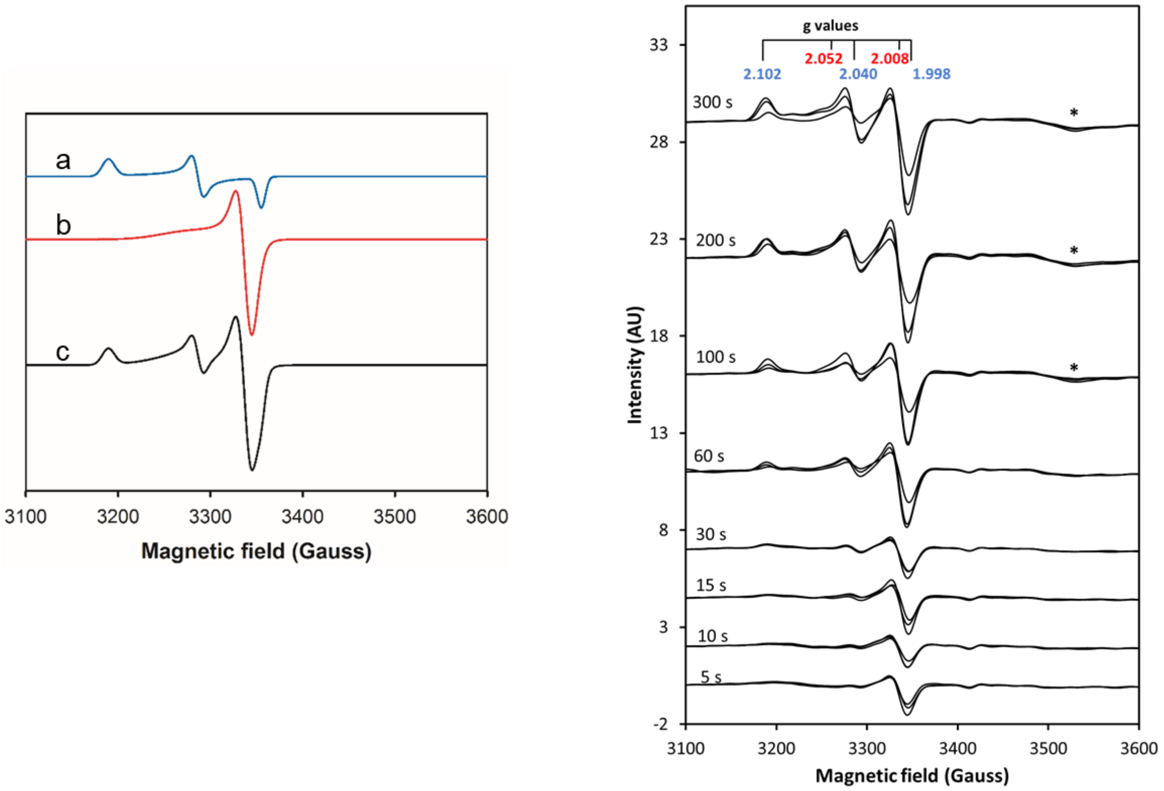

Fig. S1. Overview of simulated and experimental EPR spectra. Left panel: Simulated spectra of (a) **Hox**; (b) **Hox-CO**; (c) the total simulation of the spectrum of apo-HydA / $[2Fe]^{adt}$ after an incubation period of 200 seconds by linear combination of spectra in (a) and (b). The relative contributions of the **Hox** and **Hox-CO** EPR signals to the resultant spectrum were found to be 0.47 and 0.53, respectively. (cf. Fig. 2, bottom panel). *g*-values used for the simulation: **Hox**: g_1=2.102, g_2=2.040, g_3=1.998; **Hox-CO**: g_1=2.052, g_2=2.008, g_3=2.008. The simulation was carried out in EasySpin 5.2 [3], run as a toolbox in Matlab 2016b (the MathWorks, Inc., Natick, Massachusetts). Right panel: H-cluster assembly monitored by EPR spectroscopy. Spectra recorded for mixtures of apo-HydA1 and $[2Fe]^{adt}$ incubated for increasing mixing periods (indicated in the figure). For each time-point three samples were prepared and their respective spectra are overlaid. Reported *g*-values for **Hox** (blue) and **Hox-CO** (red) are indicated; a feature at g = 1.91 (asterisks) is attributable to a reduced iron-sulfur cluster (see main text).

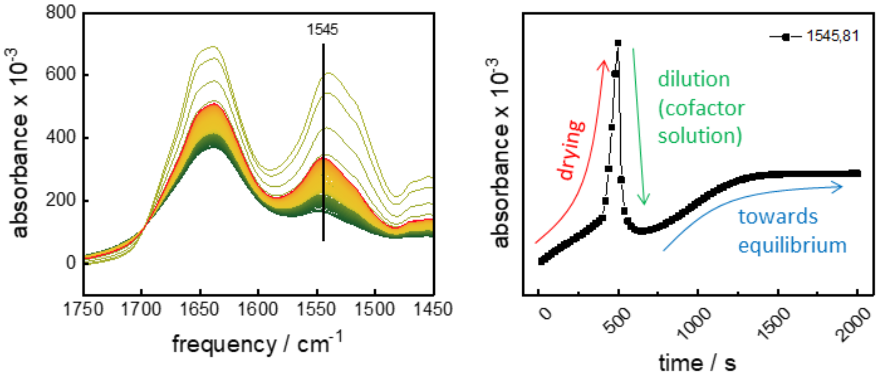

Fig. S2. Protein film hydration monitored by ATR FTIR spectroscopy. Left panel: FTIR spectra shown in Fig. 3 and Fig. S4 were normalized according to the amplitude of the amide II protein band at 1545 cm^{-1}, which varied due to variations in the hydration level of the HydA1 protein film. Right panel: Amplitude of the amide II band during the protein/complex mixing experiment. Increasing film dehydration (drying) prior to $[2Fe]^{adt}$ addition causes an increase in the protein band signals, addition of the $[2Fe]^{adt}$ solution onto the film results in a transient drop of the band amplitudes due to the film hydration, and increasing dehydration at longer incubation periods finally results in constant IR intensities of protein and H-cluster bands.

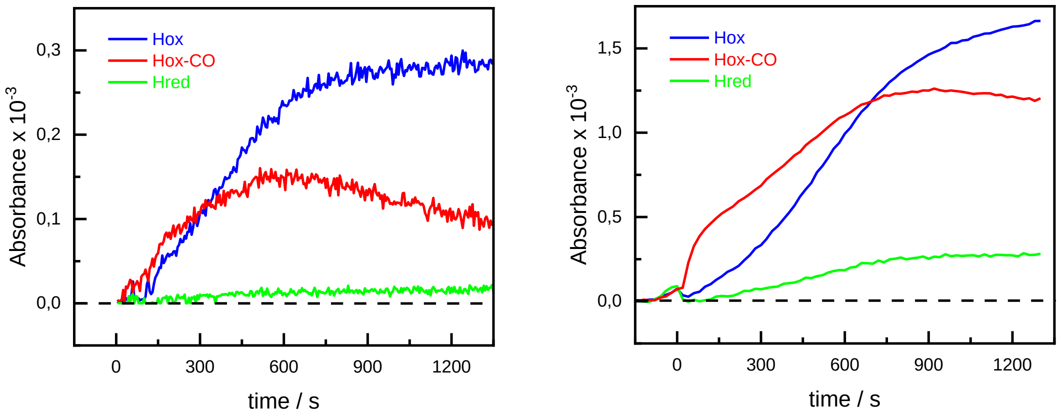

Fig. S3. Influence of the $[2Fe]^{adt}$ concentration on H-cluster assembly. Maturation of apo-HydA1 with ~0.8 µM (left panel) and ~80 µM (right panel) $[2Fe]^{adt}$ solution. Regardless of $[2Fe]^{adt}$ concentrations the same order of events was observed, i.e. **Hox-CO** formation preceding **Hox** appearance. The overall cofactor formation yield is much larger in the presence of ~80 µM $[2Fe]^{adt}$ solution, but apparent faster **Hox** formation was observed with ~0.8 µM $[2Fe]^{adt}$ solution; (marker bands: **Hox-CO**, 2012 cm^{-1}; **Hox**, 1940 cm^{-1}; **Hred**, 1891 cm^{-1}).

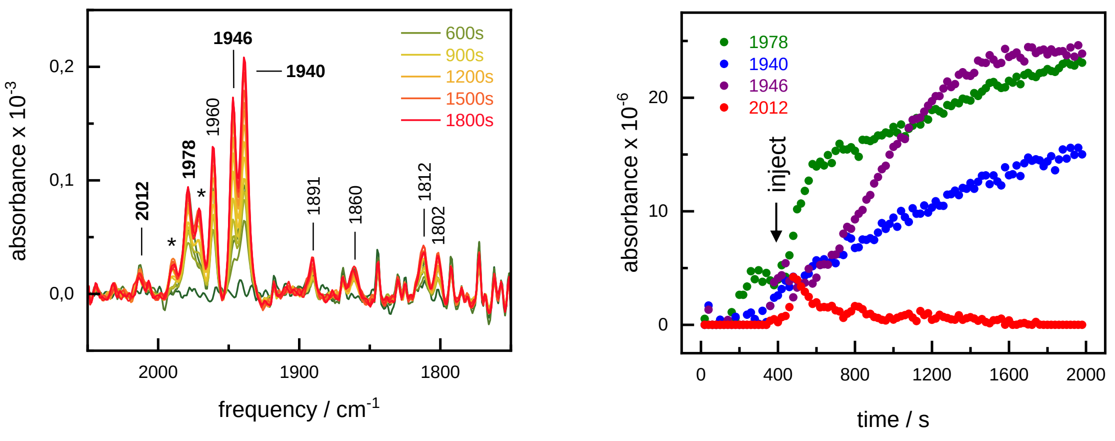

Fig. S4. H-cluster assembly under reducing conditions. 78 μM [2Fe]^{adt} was added to a film of 500 μM apo-HydA1 (pH 8) in the presence of 1 mM sodium dithionite (DT) and the reaction was monitored by the appearance of H-cluster specific bands in the CO vibrations region. Left panel: FTIR spectra in the CO region at selected mixing periods. Spectra are normalized at the amide II band at 1545 cm^{-1} (not shown). Sharp bands are assigned to water vapor. Right panel: Appearance of specific H-cluster states as a function of time as derived from respective marker bands in the right panel (**Hox-CO**, 2012 cm^{-1}; **Hox**, 1940 cm^{-1}; **HoxH**, 1946 cm^{-1}; **Hhyd**, 1978 cm^{-1}). Bands marked with an asterisk in the left panel (*i.e.*, 1988 and 1971 cm^{-1}) hint at the existence of **Htrans**.

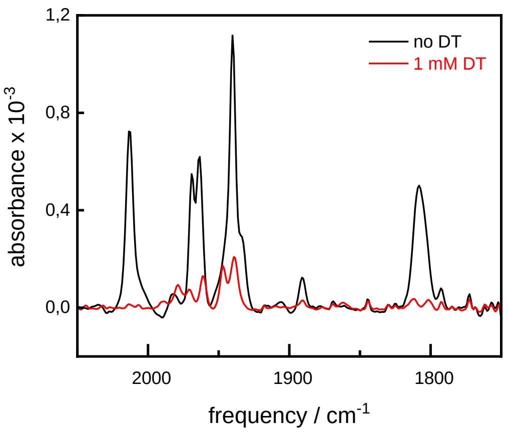

Fig. S5. Effect of reductant on H-cluster assembly (comparison). H-cluster assembly efficiency in the presence (red spectrum) or absence (black spectrum) of the reductant sodium dithionite (DT). Spectra were recorded after a apo-HydA1/[2Fe]^{adt} mixing period of 1900 s (Figs. 3 and S4) for identical protein concentrations with or without pre-treatment of the apo-HydA1 film with DT.

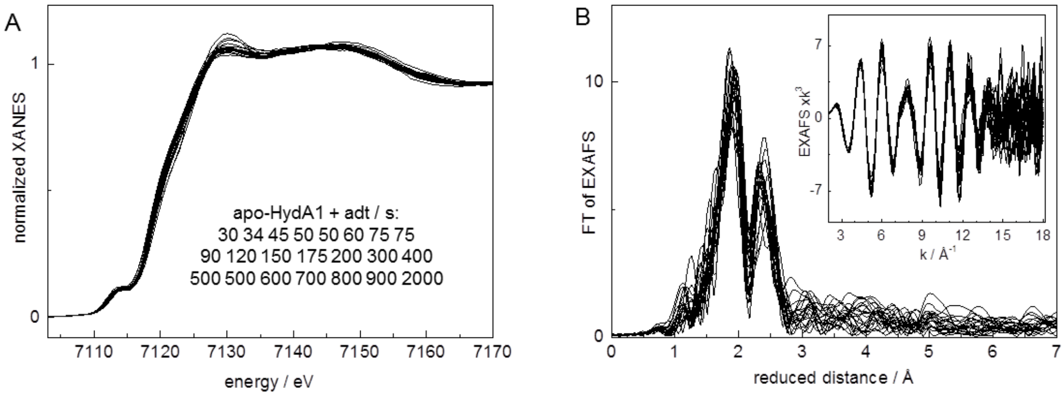

Fig. S6. Fe XAS spectra of apo-HydA1/[2Fe]^{adt} mixtures. Mixtures were incubated prior to freezing for the indicated approximate time periods. 22 spectra from two series of samples are overlayed. (A) XANES spectra. (B) Fourier-transforms of EXAFS spectra in the inset.

References

1 R. Kositzki, S. Mebs, N. Schuth, N. Leidel, L. Schwartz, M. Karnahl, F. Wittkamp, D. Daunke, A. Grohmann, U.-P. Apfel, F. Gloaguen, S. Ott and M. Haumann (2017) Dalton Trans 46:12544-12557

2 J. Esselborn, N. Muraki, K. Klein, V. Engelbrecht, N. Metzler-Nolte, U. P. Apfel, E. Hofmann, G. Kurisu and T. Happe (2016) Chem Sci 7:959-968

3 S. Stoll, A. Schweiger (2006) J. Magn. Reson. 178: 42-55
